# Precondition of right frontal region with anodal tDCS can restore the fear memory impairment induced by ACPA in male mice

**DOI:** 10.17179/excli2016-693

**Published:** 2017-01-02

**Authors:** Fariborz Manteghi, Mohammad Nasehi, Mohammad-Reza Zarrindast

**Affiliations:** 1 Institute for Cognitive Science Studies (ICSS), Tehran, Iran; 2 Cognitive and Neuroscience Research Center (CNRC), Tehran Medical Sciences Branch, Islamic Azad University, Tehran, Iran; 3 Department of Pharmacology, School of Medicine, Tehran University of Medical Sciences, Tehran, Iran

**Keywords:** tDCS, ACPA, preconditioning, fear memory, memory enhancement, learning acceleration

## Abstract

Fear memory and learning cause behavioural patterns such as fight or flight responses, which increase survival probability, but unfit processing of fear memory and learning can lead to maladaptive behaviours and maladies such as phobias, Post-Traumatic Stress Disorder (PTSD) and anxiety disorders. The growing prevalence of these maladies shows the need to quest novel methods for their treatment. We used anodal transcranial direct current stimulation (tDCS) on the right frontal region as a precondition neuromodulator and arachidonylcyclopropylamide (ACPA), a selective CB1 cannabinoid receptor agonist, as a fear memory impairing agent to assess their effects on contextual and auditory fear conditioning (reliable model for fear studies). Right frontal anodal tDCS (0.2 mA for. 20 minutes) 24 hours before the train did not alter contextual and auditory learning and memory in short-term (24 hrs after the training phase). Moreover, intraperitoneal pre-train injection of ACPA (0.1 mg/kg) alone, decreased both contextual and auditory learning and memory in short- but not long-term. Right frontal anodal tDCS improved short-term contextual fear memory in subthreshold doses of ACPA. On the other hand, right frontal anodal tDCS in long-term improved (lower doses of ACPA) and restored (higher doses of ACPA) both fear memories. These findings showed that, aforementioned approach could cause durable learning and memory improvements. Also this combined modality could be useful for fear extinction training and maladies which inflict amnesia.

## Introduction

Fear memory and learning are essential abilities, that animals and humans benefit from, to evade dangers of their environment. When these fear responses become excessive (by unfit processing of an aversive stimulus), maladies like panic disorders, phobias, PTSD and anxiety disorders may occur. Statistics have shown that 4.7 % of adult Americans suffer from a life time panic disorder, also 12.1 % have life time social phobias and 6.8 % bear life time PTSD; moreover, 28.8 % endure any kind of anxiety disorder. These prevalence and their treatment expenses, for instance during. 2010 in Europe alone, an estimated of 74.4 billion Euros were spent to treat anxiety disorders (Layton and Krikorian, 2001[21]; Olesen et al., 2012[44]), motivated us to do more research in this field to find a new effective treatment.

The fear causing stimulus is evaluated by endocannabinoid signalling and by this evaluation, the proper behavioural responses, which are necessary for existence, homeostasis and stress resilience are exerted, any flaw in this signalling system can lead to the psychiatric disorder (Lutz et al., 2015[26]). Endocannabinoids also have vital roles in maintaining emotional homeostasis (Marco and Viveros, 2009[28]) as well as excluding the unpleasant memories (Marsicano et al., 2002[32]). Most of endocannabinoid brain signalling are through G-protein coupled CB1 receptors (Caballero and Tseng, 2012[6]), which high concentration of them are present in prefrontal cortex, hippocampus and amygdala. It seems that these receptors are important in modulating learning and memory (Castellano et al., 2003[7]; Marsicano and Kuner, 2008[31]; Marsicano et al., 2002[32]), also CB1 agonists can help recalling and consolidating fear extinction memory (Abush and Akirav, 2010[1]; Das et al., 2013[11]; Rabinak et al., 2013[51]). Moreover, CB1 and CB2 cannabinoid receptor agonist (WIN55212-2) in infralimbic region facilitates the fear extinction;cannabinoid receptors are also involved in adaptation to repellent conditions (Lin et al., 2009[24]), furthermore, the intraperitoneal injection of WIN55212-2 can facilitate fear extinction and can also improve spatial memory (Pamplona et al., 2006[45]). Also, CB1 receptor inadequacy induce fear extinction deterioration (Marsicano et al., 2002[32]). Infusion of CP55,940 a ppotent, non-selective cannabinoid receptor agonist, in both infralimbic and CA1 areas in rats brain, induces the long-lasting disruption of fear memory reconsolidation and causes the reduction in freezing response (Santana et al., 2016[53]). Besides, we can boost the synaptic plasticity in both rodents and humans by stimulating the CB1 receptors (Mori et al., 2014[37]), in addition, the CB1 receptor deficiency increases the contextual fear memory and also can change hippocampal synaptic plasticity, revealing the essential role of endocannabinoid signalling in learning and memory (Jacob et al., 2012[17]). Modulation of endocannabinoid (eCB) system can be considered as a novel direction towards finding a treatment for anxiety-related disorders with least adverse effects of cannabinoids (Chhatwal et al., 2005[9]; Patel and Hillard, 2006[47]). Other modalities like tDCS can be used to modulate endocannabinoids. 

tDCS is a neuromodulatory technique which has been widely used over the past 17 years (Fregni et al., 2015[13]). This popularity is for being non-invasive and painless, as well as having no or minimum side effects along with its affordability and operational simplicity (Bastani and Jaberzadeh, 2012[4]; Nitsche and Paulus, 2000[43]). tDCS exerts its modulatory properties through facilitating (depolarization) or inhibiting (hyperpolarization) as well as adjusting excitability (increasing/decreasing cortical excitability) of nerve cell membrane in target brain regions, therefore by priming the behavioural systems, tDCS can produce agreeable changes in the cognitive systems (Miniussi et al., 2013[35]). Also cathodal stimulation of the left Dorso-Lateral Prefrontal Cortex (DLPFC) in the humans, have inhibitory effect on fear memory consolidation (Asthana et al., 2013[3]). It seems that not only polarity but also current flow direction affect the tDCS stimulation outcomes, for instance a study proposed that specific montage for tDCS (anode over right prefrontal region while cathode over left supraorbital region) increased fear memories in humans, probably by affecting the prefrontal cortex-amygdala circuit (Mungee et al., 2014[38]). In another study anodal tDCS over the left DLPFC significantly decreased attentional bias for social threat;this attentional bias is related to social anxiety disorder (SAD), hence the authors recommended tDCS as an advanced method to investigate SAD mechanisms (Heeren et al., 2016[15]), furthermore, in other study tDCS over DLPFC caused significant reduction of vigilance toward threatening stimulus; which was as effective as anxiolytic treatments. These results showed that tDCS over DLPFC could strongly change the processing of threat data (Ironside et al., 2016[16]). All above findings highlighted the importance of prefrontal (prominent brain region for emotion and attention processing) activity modulation for treating fear related diseases.

The aim of this study was to investigate the effects of tDCS on fear memory responses caused by selective cannabinoid CB1 receptor agonist (ACPA), both in short- and long-term. Recently, these two modalities have been considered seriously as a novel treatment of fear-related conditions. 

## Materials and Method

### Animals

This study was designed in such a way to lessen the number of subjects and their suffering (Nasehi et al., 2016[39]). Subjects were sixty four male NMRI mice their suffering (Nasehi et al., 2016[39]). Subjects were sixty-four male NMRI mice weighting 28-32 gr and were acquired from animal house of the Institute for Cognitive Science Studies; Tehran - Iran. The study procedures were performed in the room temperature (22 ± 2° C) and at 12 h/12 h light/dark cycle (lights turned on at 7:00 A.M.), also subjects had free access to food and water. One hour prior to starting experiment, subjects were transferred to experiment room undisturbed.

The experiment procedures were approved by Tehran University of Medical Sciences ethical committee and were in accordance with the National Institutes of Health Publication No.85-23, revised. 2010, Animal Care and Use of Laboratory Animals guidelines.

### Drug

ACPA (Arachidonylcyclopropylamide; Tocris, Cookson Ltd., UK), a selective cannabinoid CB1 receptor agonist, was dissolved in anhydrous ethanol at a concentration of 5 mg/ml and was diluted by normal saline to achieve the required doses. The drug was injected intraperitoneally at doses of 0.01, 0.05 and 0.1 mg/kg. 

### Surgery

Ketamine (50 mg/kg) and xylazine (5 mg/kg) were injected intraperitoneally to anesthetize the subjects. Their skulls were uncovered after they were secured in stereotaxic equipment, then anodal electrode with a 2.1 mm internal diameter and capacity to provide 3.5 mm^2^ effective contact area (Pedron et al., 2014[49]) was fixed over the right frontal region by dental cement. The corresponding area for right frontal region was 1 mm anterior and 1 mm to the right of Bregma (Paxinos and Franklin, 2004[48]). After surgery, all subjects were allowed five days of recovery time.

### Transcranial brain stimulation

In order to avoid anesthetizing the subjects during tDCS, a custom made device was used to confine them and therefore exclude the interference effects of anesthetic drugs on stimulation. The anode was planted over the right frontal region, and the cathode which was 9.5 cm^2^ carbon rubber in the soaked sponge cover was placed under the subject's chest. This setup allowed us to avoid electrical current diversion (Liebetanz et al., 2009[23]) and to maintain the safety and efficiency of the stimulation. Active Dose II unit (Activatek company-Taiwan) was used for tDCS, moreover, all groups were stimulated at the same time of the stimulation day, which was one day before fear conditioning train. Control group received sham tDCS. tDCS group subjects were sacrificed, after that their brain were excavated and sliced by vibroslicer and then the acquired slices were examined under microscope, no abnormalities were noticed.

### Fear conditioning 

Fear conditioning method was achieved on basis of our earlier studies, (Nasehi et al., 2016[40][41]; Shoji et al., 2014[56]). An acoustic insulated chamber with the dimensions of 55 × 53× 67 cm^3^, which illuminated by 24 watts bulb and equipped with two speakers and a video camera was used for fear conditioning. In the training phase, subjects were placed in a transparent 25 cm^3^ plexiglass container with a shock mesh on its floor. The subjects were permitted to search the area freely for 120 seconds, after that, the tone conditional stimulus (CS) with a 4 kHz frequency and 35 dB intensity was transmitted for 30 seconds, and during its last 2 seconds the subjects were exposed to a foot shock with 1 mA intensity and 50 Hz frequency as an unconditional stimulus (US). The subjects were allowed to stay in the container for extra 30 seconds to prevent their handling, affecting the associative memory, which has just been formed. In order to clean the container and rubbing out any cues, a 70 % ethanol solution was used after completion of the training and test, for each subject.

In contextual associative memory test, 24 hours after training, subjects were put in the same train area, for 300 seconds with no CS and US exposure. For auditory associative memory, one hour after contextual associative memory test, subjects were put in a completely different place to form a new context, the tone (CS) played for 3 minutes. The subjects' responses to both tests were analyzed and scored by someone who had no information about the subject's categories. Parameters which are related to fear and anxiety behaviors (Latency to freezing, freezing, grooming and rearing times) were examined. 

### Experimental design

Each group consisted of eight mice and was tested twice with fourteen days, time interval. ACPA was administered intraperitoneally (10 ml/kg) 15 minutes before training phase, while control groups received vehicle. Twenty four hours after training, contextual and auditory tests were performed (with one hour interval).

### Data analysis

SPSS version. 19 was used for statistical analysis, one- or two-way analysis of variance (ANOVA) and repeated measure were used to evaluate our results. In combination studies, two-way ANOVA was performed with four levels (0, 0.01, 0.05 and 0.1 mg/kg) of the ACPA as intra-group, and two levels of, without tDCS and with tDCS as inter-group factors. The repeated measure was performed between test and retest. We implemented tukey post hoc test for dual group assessments. The level of significancy was P < 0.05 in all experimental results. 

## Results

### Short-term and long-term effects of ACPA on the context-and-tone related fear memory acquisition

**Figure 1:**
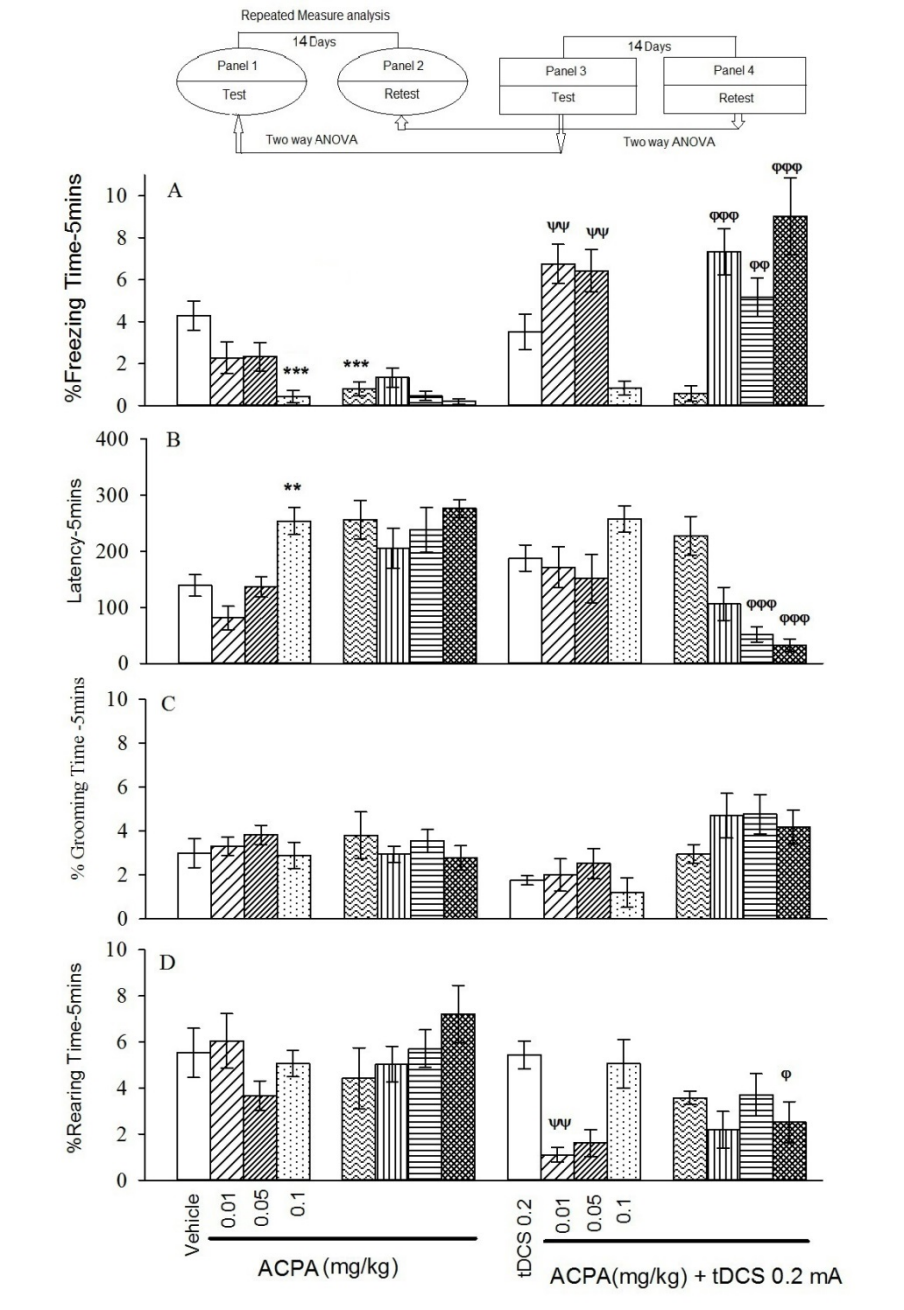
Short-term and long-term effects of ACPA in absence or presence of right frontal tDCS on memory acquisition in the context-dependent fear conditioning. The animal received tDCS one day before training and vehicle (10 ml/kg) or different doses of ACPA (0.01, 0.05 and 0.1 mg/kg) 15 minutes prior to training. The test session was performed 1 and 15 days after the training (14 days interval). % freezing (A), latency to the freezing (B), % grooming time (C) and % rearing time (D). Values are expressed as mean ± S.E.M (n = 8 in each group). ** P < 0.01 and *** P < 0.001 different from control group in the panel 1. Ψψ P < 0.001 different from the control group in the panel 3. Φ P < 0.05, φφ P < 0.01 and φφφ P < 0.001 different from control group in the panel 4.

The repeated measure and Tukey's post hoc analysis indicated that at all administered doses of ACPA only 0.1 mg/kg reduced % freezing time and increased latency to the freezing in the short-term, meanwhile the % grooming time and % rearing time showed no significant differences for contextual fear memory acquisition in both short- and long-term (Figure 1, panels 1 and 2(Fig. 1)).

**Figure 2:**
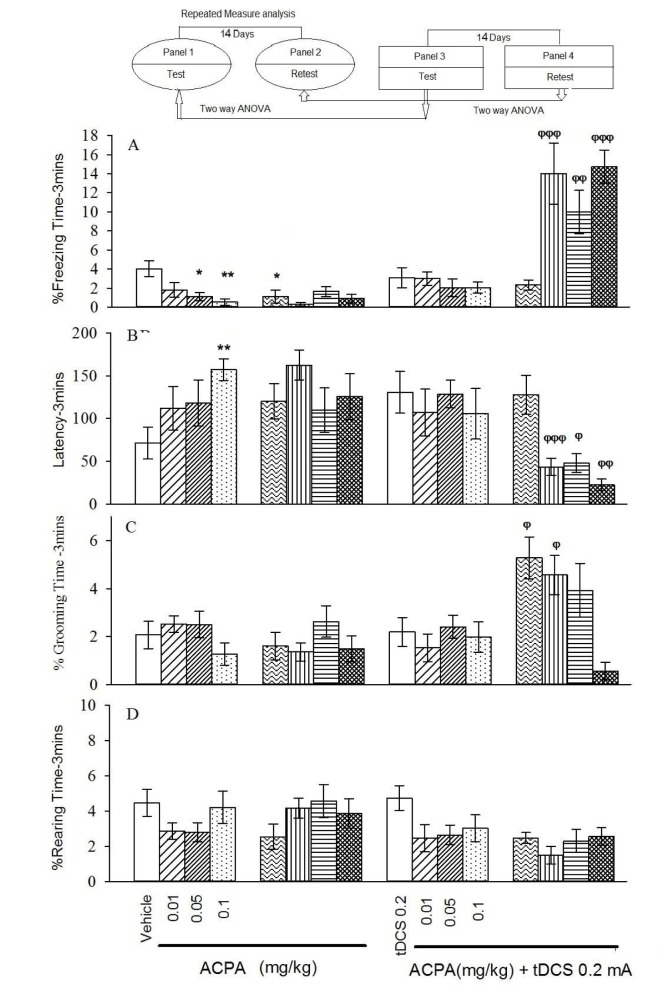
Short-term and long-term effects of ACPA in absence or presence of right frontal tDCS on memory acquisition in the auditory-dependent fear conditioning. The animals received tDCS one day before training and vehicle (10 ml/kg) or different doses of ACPA (0.01, 0.05 and 0.1 mg/kg) 15 minutes prior to training. The test session was performed 1 and 15 days after the training (14 days interval). % freezing (A), latency to the freezing (B), % grooming time (C) and % rearing time (D). Values are expressed as mean ± S.E.M (n = 8 in each group). * P < 0.05 and ** P < 0.01 different from control group in the panel 1. Φ P < 0.05, φφ P < 0.01 and φφφ P < 0.001 different from control group in the panel 4.

In auditory memory acquisition, 0.05 and 0.1 mg/kg doses of ACPA showed decreased % freezing time and increased latency to freezing in short-term (Figure 2, panels 1 and 2(Fig. 2)). % Grooming time and % rearing time did not change for both short- and long-term. 

To sum up, the data showed that ACPA had only short-term effect on both contextual and auditory fear memories and had no effect on grooming and rearing behaviors. All statistical analysis data have been shown in the Table 1(Tab. 1).

### Effects of right frontal anodal tDCS on the ACPA-induced behaviors in short-term

The two-way ANOVA and post hoc analysis showed that in contextual fear memory (Figure 1, panel 3(Fig. 1)), right frontal anodal tDCS in subthreshold doses of ACPA (0.01 and 0.05 mg/kg) increased % freezing time significantly, but it had no effect on effective dose of ACPA (0.1 mg/kg). 

Similar analysis indicated that right frontal anodal tDCS had no effect on latency to freezing and % grooming time (Figure 1, panel 3(Fig. 1)) for the contextual states induced by ACPA.

Furthermore, right frontal anodal tDCS decreased % rearing time induced by ACPA (0.01 mg/kg) in the contextual (Figure 1, panel3(Fig. 1)) states.

In auditory fear memory no significant changes were seen for all evaluated parameters in short-term. 

In conclusion all data indicated that right frontal anodal tDCS in applied intensity did not affect both contextual and auditory fear memory formation by itself. Although this intervention did not restore memory acquisition deficit induced by effective dose of ACPA (0.1 mg/kg), but it did interestingly enhance memory in subthreshold doses of ACPA (0.01 and 0.05 mg/kg) in short-term. All statistical analysis data have been shown in the Table 2(Tab. 2).

### Effects of right frontal anodal tDCS on the ACPA-induced behaviors in long-term

#### Contextual effects

Two-way ANOVA followed by post hoc analysis showed that right frontal anodal tDCS increased % freezing time in all ACPA doses (0.01 ,0.05 and 0.1 mg/kg), while decreased latency to freezing at 0.05 and 0.1 mg/kg doses. tDCS decreased % rearing time at 0.1 mg/kg dose, while did not alter % grooming time (Figure 1, panel 4(Fig. 1)) in long-term state. 

In conclusion right frontal anodal tDCS alone, in long term is not effective in all investigated behaviours. It also noted that right frontal anodal tDCS increased % freezing time and decreased latency to freezing and % rearing time, while did not alter % grooming time changes induced by ACPA.

#### Auditory effects

Two-way ANOVA followed by post hoc analysis showed that right frontal anodal tDCS increased % freezing time and decreased latency to freezing (in all administered doses of ACPA), but it increased % grooming time (at dose of 0.01 mg/kg), while it did not alter % rearing time in the long-term state (Figure 2, panel 4(Fig. 2)). 

It is noteworthy that right frontal anodal tDCS in long-term increased % grooming time, but did not alter any other behaviours by itself. Moreover, this intervention not only could restore ACPA induced amnesia (0.1 mg/kg) but also could enhance memory formation in subthreshold doses of ACPA (0.01 and 0.05 mg/kg). All statistical analysis data have been shown in the Table 3(Tab. 3).

## Discussion

### ACPA effects on fear memory formation

This study might be the first study that investigated long-term effects of ACPA on fear memory formation. Our data showed that pre-train injection of ACPA (i.p.) impaired contextual (0.1 mg/kg) and auditory (0.05, 0.1 mg/kg) fear memories in short-term (in line with earlier studies), but in long-term no effect was seen in all administered doses of ACPA. It has been shown that the cannabinoids caused inhibition of amygdala neurons firing during formation of short-term memory (Wilson and Nicoll, 2002[59]) and as one of the well-known and accepted categories of retrograde messengers in the brain cannabinoids had numerous roles on short-term regulation of synaptic transmission (Alger, 2002[2]). Also it has been reported that CB1 mediated fear inhibition through GABA and GABAergic plasticity (Kamprath et al., 2011[18]) moreover, the functional interaction between CB1 and cholecystokinin B receptor (CCKBR) which were closely located in the basolateral amygdala (BLA is a critical emotional regulation region) had a vital role in fear extinction processes (Bowers and Ressler, 2015[5]). However, some studies indicated that ACPA impairing effects on emotional memory could be via activation and deactivation of BLA serotonin 5-HT3 and 5-HT4 receptors (Chegini et al., 2014[8]) and also could be related to NMDA receptors activation in central amygdala (Ghiasvand et al., 2011[14]).

### tDCS effects on fear memory formation

According to our results right frontal anodal tDCS (0.2 mA for 20 min) in the short-term had no significant effect on fear memory formation (contextual and auditory), but conversely in the long-term enhanced both fear memories. Furthermore, grooming and rearing behaviors as anxiogenic indices did not change, so we could say that tDCS protocol had no anxiogenic effects.

There were studies showed that tDCS could enhance glutamatergic plasticity in both animals and humans directly [via modulation of neuronal calcium inflow (Nitsche et al., 2003[42])] and indirectly [via reducing GABA (Stagg et al., 2009[58])]. It was suggested that tDCS caused neuroplasticity for maintaining homeostasis (Medeiros et al., 2012[33]) by regulating variety of neurotransmitters such as dopamine, acetylcholine, and serotonin (Kuo et al., 2007[19], 2008[20]; Monte-Silva et al., 2009[36]).

Our findings showed that long-term memory enhancement occurred as a result of lasting LTP and durable learning and memory enhancement. This finding was coherent with earlier studies that have shown durable hippocampal synaptic plasticity could be reached by using tDCS (Rohan et al., 2015[52]) and even one exposure could be sufficient for learning new associative memory and behavioral patterns (Pasupathy and Miller, 2005[46]; Schultz et al., 2003[54]).

### tDCS plus ACPA effects on fear memory formation

The effect of preconditioning with tDCS on fear memory responses induced by ACPA was also investigated. tDCS restored the contextual amnesia induced by subthreshold doses of ACPA (0.01 and 0.05 mg/kg) in short-term, while improved fear memory formation by all applied doses of ACPA in long-term in both contextual and auditory fear memories formation. % Grooming and % rearing times mostly showed no significant changes (except for % grooming time in long-term auditory fear memory in tDCS alone and in tDCS with 0.01 mg/kg ACPA groups), thus we could conclude that our method did not cause anxiety-like behaviors.

Earlier findings suggested that synaptic plasticity was the underlying mechanism for different types of memory (Malenka and Nicoll, 1999[27]), also the endocannabinoids involvement in short-term (Wilson and Nicoll, 2001[60]) and long-term synaptic plasticity were shown (Melis et al., 2004[34]; Zygmunt et al., 2000[62]). Our results could be explained by Lin et al. (2011[25]) findings that high-frequency (HFS) and theta-burst stimulations induced long-term depression of ȣ-aminobutyric acid (GABA) transmission by endocannabinoids, this induction intensely affected the excitatory postsynaptic potential through metaplasticity. Furthermore, Cui and others (2015[10]) found out that eCB is a bidirectional plasticity system which could cause rapid learning, depending on synaptic state. On the other hand, changing the state of synapse by preconditioning with tDCS can alter the expected effects of other modalities such as rTMS (Quartarone et al., 2005[50]; Siebner et al., 2004[57]). 

Different results in short-term contextual and auditory fear memories could be related to various pathways of these two fear memories, for instance vital involvement of dorsal hippocampus in contextual fear memory formation and retrieval (Maren et al., 2013[30]; Zhou et al., 2016[61]) and also dissimilar amygdala nuclei participation in the contextual [basolateral and basomedial amygdala (Maren and Fanselow, 1995[29])] and in the auditory [lateral amygdaloid nucleus (Doron and Ledoux, 1999[12]; LeDoux et al., 1991[22])] fear memories.

Our data suggested that fear circuit had homeostatic system. It has shown that homeostatic systems follow regulatory mechanisms for preserving stability by keeping neuron activity in functional dynamic range (Sejnowski, 1977[55]). Understanding these mechanisms which can broaden the usage of tDCS in clinic needs more in-depth investigation.

## Notes

Mohammad Nasehi and Mohammad-Reza Zarrindast (Department of Pharmacology, School of Medicine, Tehran University of Medical Sciences, Tehran 13145-784 Iran; Tel:+9821-99881118-20, Fax:+9821-99881117, email: zarinmr@ams.ac.ir) contributed equally as corresponding authors.

## Role of the funding source

This study was supported by the Cognitive and Neuroscience Research Center (CNRC), Tehran Medical Sciences Branch, Islamic Azad University, Tehran, Iran. 

## Conflict of interest

The authors do not declare any conflict of interest. 

## Acknowledgement

The authors would like to express their appreciation to Solene Pedron, Maryam Farrahizadeh and Davoud Mahmoudzadeh Shahanaghi for their valuable help.

## Figures and Tables

**Table 1:**
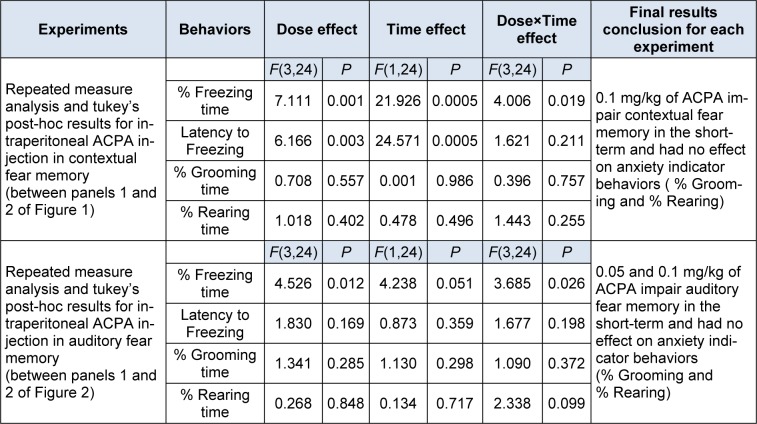
Repeated measure analysis with P values for the effect of ACPA on fear memory

**Table 2:**
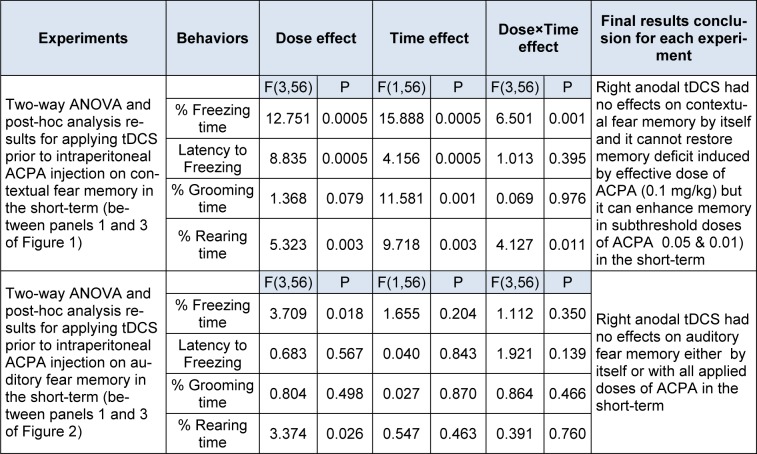
Two-way ANOVA results for the effect of tDCS prior to ACPA injection on fear memory in short-term

**Table 3:**
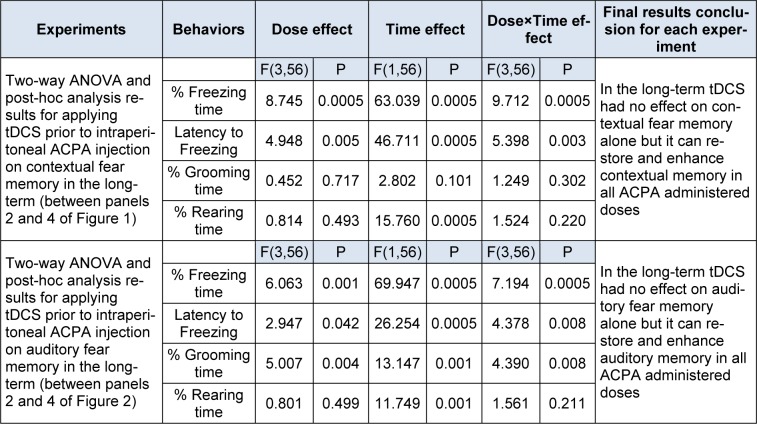
Two-way ANOVA results for the effect of tDCS prior to ACPA injection on fear memory in long-term

## References

[1] Abush H, Akirav I (2010). Cannabinoids modulate hippocampal memory and plasticity. Hippocampus.

[2] Alger BE (2002). Retrograde signaling in the regulation of synaptic transmission:focus on endocannabinoids. Progr Neurobiol.

[3] Asthana M, Nueckel K, Mühlberger A, Neueder D, Polak T, Domschke K (2013). Effects of transcranial direct current stimulation on consolidation of fear memory. Front Psychiatry.

[4] Bastani A, Jaberzadeh S (2012). Does anodal transcranial direct current stimulation enhance excitability of the motor cortex and motor function in healthy individuals and subjects with stroke:a systematic review and meta-analysis. Clin Neurophysiol.

[5] Bowers ME, Ressler KJ (2015). Interaction between the cholecystokinin and endogenous cannabinoid systems in cued fear expression and extinction retention. Neuropsychopharmacology.

[6] Caballero A, Tseng KY (2012). Association of cannabis use during adolescence, prefrontal CB1 receptor signaling, and schizophrenia. Front Pharmacol.

[7] Castellano C, Rossi-Arnaud C, Cestari V, Costanzi M (2003). Cannabinoids and memory;animal studies. Curr Drug Targets CNS Neurol Disord.

[8] Chegini HR, Nasehi M, Zarrindast MR (2014). Differential role of the basolateral amygdala 5-HT3 and 5-HT4 serotonin receptors upon ACPA-induced anxiolytic-like behaviors and emotional memory deficit in mice. Behav Brain Res.

[9] Chhatwal JP, Davis M, Maguschak KA, Ressler KJ (2005). Enhancing cannabinoid neurotransmission augments the extinction of conditioned fear. Neuropsychopharmacology.

[10] Cui Y, Paille V, Xu H, Genet S, Delord B, Fino E (2015). Endocannabinoids mediate bidirectional striatal spike‐timing‐dependent plasticity. J Physiol.

[11] Das RK, Kamboj SK, Ramadas M, Yogan K, Gupta V, Redman E (2013). Cannabidiol enhances consolidation of explicit fear extinction in humans. Psychopharmacology.

[12] Doron NN, Ledoux JE (1999). Organization of projections to the lateral amygdala from auditory and visual areas of the thalamus in the rat. J Comp Neurol.

[13] Fregni F, Nitsche M, Loo C, Brunoni A, Marangolo P, Leite J (2015). Regulatory considerations for the clinical and research use of transcranial direct current stimulation (tDCS): review and recommendations from an expert panel. Clin Res Regul Aff.

[14] Ghiasvand M, Rezayof A, Zarrindast MR, Ahmadi S (2011). Activation of cannabinoid CB1 receptors in the central amygdala impairs inhibitory avoidance memory consolidation via NMDA receptors. Neurobiol Learn Mem.

[15] Heeren A, Billieux J, Philippot P, de Raedt R, Baeken C, de Timary P (2016). Impact of transcranial direct current stimulation on attentional bias for threat: a proof-of-concept study among individuals with social anxiety disorder. Soc Cogn Affect Neurosci.

[16] Ironside M, O’Shea J, Cowen PJ, Harmer CJ (2016). Frontal cortex stimulation reduces vigilance to threat: implications for the treatment of depression and anxiety. Biol Psychiat.

[17] Jacob W, Marsch R, Marsicano G, Lutz B, Wotjak CT (2012). Cannabinoid CB1 receptor deficiency increases contextual fear memory under highly aversive conditions and long-term potentiation in vivo. Neurobiol Learn Mem.

[18] Kamprath K, Romo-Parra H, Häring M, Gaburro S, Doengi M, Lutz B (2011). Short-term adaptation of conditioned fear responses through endocannabinoid signaling in the central amygdala. Neuropsychopharmaology.

[19] Kuo MF, Grosch J, Fregni F, Paulus W, Nitsche MA (2007). Focusing effect of acetylcholine on neuroplasticity in the human motor cortex. J Neurosci.

[20] Kuo M-F, Paulus W, Nitsche MA (2008). Boosting focally-induced brain plasticity by dopamine. Cerebral Cortex.

[21] Layton B, Krikorian R (2001). Memory mechanisms in posttraumatic stress disorder. J Neuropsychiat Clin Neurosci.

[22] LeDoux JE, Farb CR, Romanski LM (1991). Overlapping projections to the amygdala and striatum from auditory processing areas of the thalamus and cortex. Neurosci Lett.

[23] Liebetanz D, Koch R, Mayenfels S, König F, Paulus W, Nitsche MA (2009). Safety limits of cathodal transcranial direct current stimulation in rats. Clin Neurophysiol.

[24] Lin HC, Mao SC, Su CL, Gean PW (2009). The role of prefrontal cortex CB1 receptors in the modulation of fear memory. Cerebral Cortex.

[25] Lin QS, Yang Q, Liu DD, Sun Z, Dang H, Liang J (2011). Hippocampal endocannabinoids play an important role in induction of long-term potentiation and regulation of contextual fear memory formation. Brain Res Bull.

[26] Lutz B, Marsicano G, Maldonado R, Hillard CJ (2015). The endocannabinoid system in guarding against fear, anxiety and stress. Nat Rev Neurosci.

[27] Malenka RC, Nicoll RA (1999). Long-term potentiation - a decade of progress?. Science.

[28] Marco E, Viveros M (2009). The critical role of the endocannabinoid system in emotional homeostasis: avoiding excess and deficiencies. Mini Rev Med Chem.

[29] Maren S, Fanselow MS (1995). Synaptic plasticity in the basolateral amygdala induced by hippocampal formation stimulation in vivo. J Neurosci.

[30] Maren S, Phan KL, Liberzon I (2013). The contextual brain:implications for fear conditioning, extinction and psychopathology. Nat Rev Neurosci.

[31] Marsicano G, Kuner R, Köfalvi A (2008). Anatomical distribution of receptors, ligands and enzymes in the brain and in the spinal cord:circuitries and neurochemistry. Cannabinoids and the brain.

[32] Marsicano G, Wotjak CT, Azad SC, Bisogno T, Rammes G, Cascio MG (2002). The endogenous cannabinoid system controls extinction of aversive memories. Nature.

[33] Medeiros LF, de Souza ICC, Vidor LP, de Souza A, Deitos A, Volz MS (2012). Neurobiological effects of transcranial direct current stimulation:a review. Front Psychiatry.

[34] Melis M, Pistis M, Perra S, Muntoni AL, Pillolla G, Gessa GL (2004). Endocannabinoids mediate presynaptic inhibition of glutamatergic transmission in rat ventral tegmental area dopamine neurons through activation of CB1 receptors. J Neurosci.

[35] Miniussi C, Harris JA, Ruzzoli M (2013). Modelling non-invasive brain stimulation in cognitive neuroscience. Neurosci Biobehav Rev.

[36] Monte-Silva K, Kuo M-F, Thirugnanasambandam N, Liebetanz D, Paulus W, Nitsche MA (2009). Dose-dependent inverted U-shaped effect of dopamine (D2-like) receptor activation on focal and nonfocal plasticity in humans. J Neurosci.

[37] Mori F, Ljoka C, Nicoletti CG, Kusayanagi H, Buttari F, Giordani L (2014). CB1 receptor affects cortical plasticity and response to physiotherapy in multiple sclerosis. Neurol Neuroimmunol Neuroinflamm.

[38] Mungee A, Kazzer P, Feeser M, Nitsche MA, Schiller D, Bajbouj M (2014). Transcranial direct current stimulation of the prefrontal cortex:a means to modulate fear memories. Neuroreport.

[39] Nasehi M, Davoudi K, Ebrahimi-Ghiri M, Zarrindast M-R (2016). Interplay between serotonin and cannabinoid function in the amygdala in fear conditioning. Brain Res.

[40] Nasehi M, Hajian M, Ebrahimi-Ghiri M, Zarrindast MR (2016). Role of the basolateral amygdala dopamine receptors in arachidonylcyclopropylamide-induced fear learning deficits. Psychopharmacology.

[41] Nasehi M, Zamanparvar M, Ebrahimi-Ghiri M, Zarrindast MR (2016). Modulation of cannabinoid signaling by amygdala alpha2-adrenergic system in fear conditioning. Behav Brain Res.

[42] Nitsche M, Fricke K, Henschke U, Schlitterlau A, Liebetanz D, Lang N (2003). Pharmacological modulation of cortical excitability shifts induced by transcranial direct current stimulation in humans. J Physiol.

[43] Nitsche M, Paulus W (2000). Excitability changes induced in the human motor cortex by weak transcranial direct current stimulation. J Physiol.

[44] Olesen J, Gustavsson A, Svensson M, Wittchen HU, Jönsson B (2012). The economic cost of brain disorders in Europe. Eur J Neurol.

[45] Pamplona FA, Prediger RD, Pandolfo P, Takahashi RN (2006). The cannabinoid receptor agonist WIN 55,212-2 facilitates the extinction of contextual fear memory and spatial memory in rats. Psychopharmacology.

[46] Pasupathy A, Miller EK (2005). Different time courses of learning-related activity in the prefrontal cortex and striatum. Nature.

[47] Patel S, Hillard CJ (2006). Pharmacological evaluation of cannabinoid receptor ligands in a mouse model of anxiety:further evidence for an anxiolytic role for endogenous cannabinoid signaling. J Pharmacol Exp Therap.

[48] Paxinos G, Franklin KB (2004). The mouse brain in stereotaxic coordinates.

[49] Pedron S, Monnin J, Haffen E, Sechter D, Van Waes V (2014). Repeated transcranial direct current stimulation prevents abnormal behaviors associated with abstinence from chronic nicotine consumption. Neuropsychopharmacology.

[50] Quartarone A, Rizzo V, Bagnato S, Morgante F, Sant'Angelo A, Romano M (2005). Homeostatic-like plasticity of the primary motor hand area is impaired in focal hand dystonia. Brain.

[51] Rabinak CA, Angstadt M, Sripada CS, Abelson JL, Liberzon I, Milad MR (2013). Cannabinoid facilitation of fear extinction memory recall in humans. Neuropharmacology.

[52] Rohan JG, Carhuatanta KA, McInturf SM, Miklasevich MK, Jankord R (2015). Modulating hippocampal plasticity with in vivo brain stimulation. J Neurosci.

[53] Santana F, Sierra RO, Haubrich J, Crestani AP, Duran JM, de Freitas Cassini L (2016). Involvement of the infralimbic cortex and CA1 hippocampal area in reconsolidation of a contextual fear memory through CB1 receptors:Effects of CP55, 940. Neurobiol Learn Mem.

[54] Schultz W, Tremblay L, Hollerman JR (2003). Changes in behavior-related neuronal activity in the striatum during learning. Trends Neurosci.

[55] Sejnowski T (1977). Statistical constraints on synaptic plasticity. J Theor Biol.

[56] Shoji H, Takao K, Hattori S, Miyakawa T (2014). Contextual and cued fear conditioning test using a video analyzing system in mice. J Vis Exp.

[57] Siebner HR, Lang N, Rizzo V, Nitsche MA, Paulus W, Lemon RN (2004). Preconditioning of low-frequency repetitive transcranial magnetic stimulation with transcranial direct current stimulation:evidence for homeostatic plasticity in the human motor cortex. J Neurosci.

[58] Stagg CJ, Best JG, Stephenson MC, O'Shea J, Wylezinska M, Kincses ZT (2009). Polarity-sensitive modulation of cortical neurotransmitters by transcranial stimulation. J Neurosci.

[59] Wilson RI, Nicoll RA (2002). Endocannabinoid signaling in the brain. Science.

[60] Wilson RI, Nicoll RA (2001). Endogenous cannabinoids mediate retrograde signalling at hippocampal synapses. Nature.

[61] Zhou H, Zhou Q, Xu L (2016). Unilateral hippocampal inactivation or lesion selectively impairs remote contextual fear memory. Psychopharmacology.

[62] Zygmunt PM, Chuang H-h, Movahed P, Julius D, Högestätt ED (2000). The anandamide transport inhibitor AM404 activates vanilloid receptors. Eur J Pharmacol.

